# A Proteomic Analysis of Discolored Tooth Surfaces after the Use of 0.12% Chlorhexidine (CHX) Mouthwash and CHX Provided with an Anti-Discoloration System (ADS)

**DOI:** 10.3390/ma14154338

**Published:** 2021-08-03

**Authors:** Stefania Bergamini, Elisa Bellei, Luigi Generali, Aldo Tomasi, Carlo Bertoldi

**Affiliations:** Department of Surgery, Medicine, Dentistry and Morphological Sciences with Transplant Surgery, Oncology and Regenerative Medicine Relevance, University-Hospital of Modena and Reggio Emilia, Via del Pozzo 71, 41124 Modena, Italy; elisa.bellei@unimore.it (E.B.); luigi.generali@unimore.it (L.G.); aldo.tomasi@unimore.it (A.T.); carlo.bertoldi@unimore.it (C.B.)

**Keywords:** chlorhexidine, anti-discoloration system, dental plaque, proteomics, one-dimensional gel electrophoresis, two-dimensional gel electrophoresis, mass spectrometry

## Abstract

Chlorhexidine (CHX) is considered the gold standard for the chemical control of bacterial plaque and is often used after surgical treatment. However, CHX employment over an extended time is responsible for side effects such as the appearance of pigmentations on the teeth and tongue; the discoloration effects are less pronounced when using a CHX-based mouthwash with added an anti-discoloration system (ADS). The aim of this study was to evaluate, using one- and two-dimensional gel electrophoresis combined with mass spectrometry, the possible proteomic changes induced by CHX and CHX+ADS in the supragingival dental sites susceptible to a discoloration effect. The tooth surface collected material (TSCM) was obtained by curettage after resective bone surgery from three groups of patients following a supportive therapy protocol in which a mechanical control was combined with placebo rinses or CHX or a CHX+ADS mouthwash. The proteomic analysis was performed before surgery (basal conditions) and four weeks after surgery when CHX was used (or not) as chemical plaque control. Changes in the TSCM proteome were only revealed following CHX treatment: glycolytic enzymes, molecular chaperones and elongation factors were identified as more expressed. These changes were not detected after CHX+ADS treatment. An ADS could directly limit TSCM forming and also the CHX antiseptic effect reduces its ability to alter bacterial cell permeability. However, Maillard’s reaction produces high molecular weight molecules that change the surface properties and could facilitate bacterial adhesion.

## 1. Introduction

Dental plaque is a complex and dynamic multispecies biofilm that develops on tooth surfaces due to the accumulation of various micro-organisms [1]. Dental plaque is formed as a biofilm whose structure and organization is directly proportional to the surface unevenness and roughness. Surface unevenness promotes bacterial colonization, protecting bacteria from the clearance forces of the salivary flow, chewing, swallowing and hygiene procedures and allows the establishment of less reversible bacteria binding. Surface roughness plays a particularly important role in de novo biofilm formation on smooth surfaces, becoming less significant over time after the biofilm ground has already been laid. Dental plaque is structurally and functionally organized, comprising hundreds of species of bacteria incorporated in an extracellular matrix of polymers deriving from the host (e.g., salivary products) and of a microbial origin [2,3]. Supragingival and subgingival plaques are the most significant biofilms in an oral cavity, contributing to the onset and progression of the most common orodental manifestations such as caries and oral and periodontal diseases such as periodontitis [4,5]. The latter is a chronic inflammatory disease caused by a persistent bacterial infection that leads to gingival inflammation, the loss of a periodontal attachment and the regression of the alveolar bone and is responsible for the development of periodontal pockets [6] and impaired dental stability [7,8]. The pathogenetic mechanisms involved in the onset of periodontal disease are not yet fully understood; an important role in periodontal damage is attributed to the chronic presence of the microbiota, which, accumulating in periodontal tissue, would trigger an excessive and persistent inflammatory response responsible for the destruction of the periodontium [9]. Individual susceptibility on a genetic basis [10,11], lifestyle (poor oral hygiene, cigarette smoking) [12] and the presence of important concomitant diseases (such as diabetes) that are not well-controlled [13] are also factors favoring periodontitis. The therapeutic approach is aimed at correcting the causes (cause-related therapy) and is based on controlling risk factors such as the microbiota and correcting lifestyles (e.g., smoking). The cause-related therapy results are particularly effective in periodontitis characterized by shallow pockets but, in general, with deeper pockets it is necessary to resort to an adjunctive therapy consisting of periodontal surgery [14]. Plaque control plays a key role in the periodontal disease treatment both with cause-related therapy and in supportive therapy after the treatment. Generally, post-surgical plaque control involves home dental care lasting from one to four weeks based on mouth rinses with chlorhexidine (CHX)-based mouthwashes (chemical control) associated with the use of a toothbrush and interdental cleaning devices (mechanical control) reintroduced at variable times after surgery [15]. To date, CHX seems to be the most effective chemical agent for plaque control even if its clinical benefits have not yet been well-defined [16]. 

The action mechanism of CHX on bacteria is still under investigation. It is believed that CHX, having a positive charge, interacts with the negative charge of membrane phospholipids resulting in an alteration of bacterial membrane permeability with the consequent leakage of low molecular weight constituents and the precipitation of cytoplasmic contents [17]. Recently, a biocidal “zombie effect” of CHX has been reported; namely, the ability of dead bacteria killed by an antimicrobial substance to act as a competent biocidal agent toward a new generation of viable bacteria [18].

However, its use does not replace mechanical therapy [19,20] and it is responsible for side effects such as tooth staining and rare anaphylactic reactions [21]. In addition, the prolonged use of CHX could lead to tolerance or even resistance to CHX in oral bacteria as well as to the development of a cross-resistance to antibiotics with consequences for infection control [22].

Currently, CHX-based mouthwashes with the addition of an anti-discoloration system (ADS) are available to contrast the onset of tooth pigmentation [23,24,25]. However, further scientific and clinical research is needed to support the safety of CHX and highlight its appropriate use [21]. 

The aim of this study was to evaluate the possible proteomic changes induced by the use of CHX and CHX+ADS in the supragingival tooth surfaces susceptible to the discoloration effect (tooth surface collected material, TSCM) by curettage. Patients undergoing periodontal surgery were divided into three parallel controlled groups. Patients in each group were asked to observe a post-surgery protocol involving the use of toothbrushes (mechanical control) combined with: (1) sterile apyrogenic water (control group), (2) 0.12% CHX mouthwash (CHX group) and (3) 0.12% CHX mouthwash plus ADS (CHX+ADS group) as a chemical plaque control.

The TSCM proteomic profiles of the three groups were evaluated before surgery (basal conditions) and after the end of early healing time (when CHX was used, or not, as a chemical plaque control), first by one-dimensional gel electrophoresis (1-DE) and then by two-dimensional gel electrophoresis (2-DE) associated with a mass spectrometry (LC-MS/MS) analysis. The identified proteins were discussed in detail considering their main molecular functions and biological processes.

## 2. Materials and Methods

### 2.1. Chemicals and Reagents

Chemicals and reagents were purchased from Merck Life Sciences Srl, Milan Italy (urea, Tris, Dithiotreitol (DTT), 2-mercaptoethanol (BME), bovine serum albumin (BSA), thiourea and 3-[(3-Cholamidopropyl)-dimethylammonio]-propane-sulfonate (CHAPS)), Roche Italia, MB, Italy (protease inhibitors), Bio-Rad Laboratories, Srl, Segrate, Milan, Italy (protein assay dye reagent, 2 X Laemmli sample buffer, ampholytes pH 3–10, Ready IPG Strip™ pH 3–10 and Precision Plus Protein™ Standards All Blue), Carlo Erba Reagents Srl, Milan, Italy (acetone, acetonitrile (ACN) and formic acid (FA), all of MS purity grade), Life Technologies Italia, MB, Italy (Precast gel Bolt™ 12% Bis-Tris Plus and MES SDS 10 X running buffer), Dentaid Srl, Bologna, Italy (mouthwashes 0.12% CHX Perio-AID^®^), Curaden HealthCare S.p.a, Saronno, Varese, Italy (Curasept CHX 0.12% + ADS) and Eurospital S.p.a, Trieste, Italy (sterile apyrogenic water).

### 2.2. Patient Recruitment and TSCM Collection

Patients diagnosed with periodontitis and candidates for periodontal resective bone therapy were selected at the Complex Dentistry and Oral-Maxillofacial Surgery Unit (University Hospital of Modena and Reggio Emilia, Italy) after the approval of the Provincial Ethics Committee of Modena (protocol nr.4406/2017, registration nr. 369/17). 

The selection was made taking into account inclusion and exclusion criteria after the completion of cause-related therapy. Candidates had to be in good systemic health and suffering from medium/moderate periodontal disease with a full-mouth plaque score (FMPS) < 25%, a full-mouth bleeding score (FMBS) < 25% and high compliance assessed during the cause-related therapy. At least one tooth with a probing pocket depth (PPD) and a clinical attachment level (CAL) of at least 4 mm in at least 2 measurements associated with at least a 1-wall intrabony defect of at least 2 mm or a bone crater had to be present. The selected experimental periodontal area (one area per patient) had to be treated with periodontal resective surgery. The exclusion criteria were age over 70 years, severe systemic diseases (such as cardiovascular disease, diabetes), alcohol abuse, pregnancy and childbirth. Patients received detailed information regarding the procedures and the purpose of the study, as required by the Helsinki protocol [26], and provided their written informed consent to participate. 

The 27 patients enrolled in the study (of both sexes and aged 40 to 70 years) were randomly divided into 3 groups, each consisting of 9 subjects. They were asked to follow different protocols for plaque control for 4 weeks, starting the day after surgery: a mechanical treatment using toothbrushes associated with 2 daily rinses for 1 min/each with 10 mL of sterile apyrogenic water (control group, C; 9 subjects; age range 40–53 years; median 52) or 10 mL of 0.12% CHX mouthwash (CHX group; 9 subjects; age range 42–70 years; median 52) or 0.12% CHX+ADS mouthwash (CHX+ADS group; 9 subjects, age range 49–70 years; median 56). Afterwards, two TSCM samples were collected from the supragingival sites, the middle and incisal thirds of dental crowns, from each patient: the first one before surgery (baseline, B) and the second one 4 weeks after the surgical treatment (T) at the end of early healing time and possible CHX use. The samples were harvested from the dental crown to collect, in particular, material more directly linked to the chemical plaque control system adopted in each group at the second time. All samples were stored at –80 °C until the proteomic analysis, which was performed on three pools for each group: C group-B and C group-T, CHX group-B and CHX group-T, CHX+ADS group-B and CHX+ADS group-T.

### 2.3. Protein Extraction from TSCM

Proteins were extracted according to the following protocol: first, the pools were incubated overnight (O/N) in a Thermomixer (Eppendorf, Milan, Italy) with 50 μL of solubilization buffer (SB) composed of 7 M urea, 2 M thiourea, 3% CHAPS, 40 mM Tris pH 8.3, 1% ampholytes pH 3–10 and protease inhibitors. Afterwards, the samples were centrifuged at 10,000× *g* for 10 min and the supernatants (samples a) were precipitated with cold acetone (1:12, *v/v*) by incubation O/N at –20 °C. The samples were then centrifuged at 14,000× *g* for 10 min at room temperature and the pellets (samples b) were first resuspended in 50 μL of rehydration buffer (RB) (composed of 6 M urea, 2 M thiourea, 4% CHAPS, 25 mM DTT and 0.2% ampholytes pH 3–10) and then were added to 50 μL of SB prior to incubation O/N at +10 °C. Finally, samples a and b of each pool were combined into a single sample. The protein assay was carried out by Bradford’s method using the protein assay dye reagent and BSA as a colorimetric solution and a standard calibrator, respectively. A spectrophotometric reading was performed at λ = 595 nm in a microplate reader (Multiskan^TM^ FC, Thermo Fisher Scientific, Waltham, MA, USA).

### 2.4. Proteomic Analysis

All pools were first subjected to 1-DE and subsequently to 2-DE analysis. 1-DE was performed under reducing conditions. Briefly, an aliquot of each pool (corresponding with 12 µg protein) was mixed with the 2 X Laemmli sample buffer containing 0.5% BME and incubated at 95 °C for 5 min. Protein separation was performed using precast gel Bolt^TM^ 12% Bis-Tris Plus and the MES SDS 1 X running buffer, setting the power supply at 100 V for the first 30 min then increasing to 200 V until the end of the electrophoretic run. 2-DE analysis was performed as earlier reported [27]. In brief, after TSCM solubilization with RB, immobilized pH-gradient (IPG) strips, pH range 3–10, 7 cm long (Ready Strip™) were employed in the first-dimension separation while mini-gradient polyacrylamide gels 8–16% were used in the second-dimension separation. Finally, protein spots, as well as protein bands obtained by 1-DE, were highlighted with a silver nitrate staining protocol as previously fully described [28]. Both 1D and 2D gel images were acquired by a calibrated densitometer (model GS-800, Bio-Rad, Hercules, CA, USA) and analyzed with specific image analysis software.

### 2.5. Protein Identification by Mass Spectrometry

Protein spots in the MW range between 25–100 kDa were subjected to an MS analysis after in-gel trypsin digestion, as already reported [29]. The protein identification was performed by an UHPLC-MS QExactive™ (ThermoScientific, Waltham, MA, USA) system, consisting of an UHPLC 3000 Ultimate System coupled to an ESI-QExactive™ Hybrid Quadrupole-Orbitrap™ mass spectrometer (LC-ESI-QO-MS/MS System). The MS analysis was carried out as previously fully described [30]. Briefly, dried samples were resuspended in 40 μL of a water/ACN/FA solution (95:3:2), then sonicated for 10 min and finally centrifuged at 12,000× *g* for 10 min prior to injection into the instrument. The protein identification was achieved by the MASCOT search engine using NextProt and SwissProt databases.

### 2.6. Statistics

All data are provided as a mean ± standard deviation (SD). The analysis of variance test (ANOVA) and Student–Newman–Keuls test (SNK) for multiple comparisons were applied for the data analysis among groups while a paired Student’s t-test was used for the data analysis within the same group, considering *p*-values < 0.05 as statistically significant.

QuantityOne 1D image analysis software (version 4.6.7, Bio-Rad) and the PDQuest 2D image analysis software program (version 7.3.1, Bio-Rad), were used to evaluate the protein bands revealed by 1-DE and the protein spots detected by the 2-DE analysis, respectively. The signal intensity of the protein bands and spots were expressed as optical density (OD). In order to correct the variability due to the staining procedure, band volumes were normalized as percentage of the total OD of all the bands detected in the gel while 2D gel images were normalized by a filtration process. Protein spots with an expression variance > 1.5-fold were selected as significantly different between the studied groups.

## 3. Results

### 3.1. 1-DE Analysis

**Figure 1:**
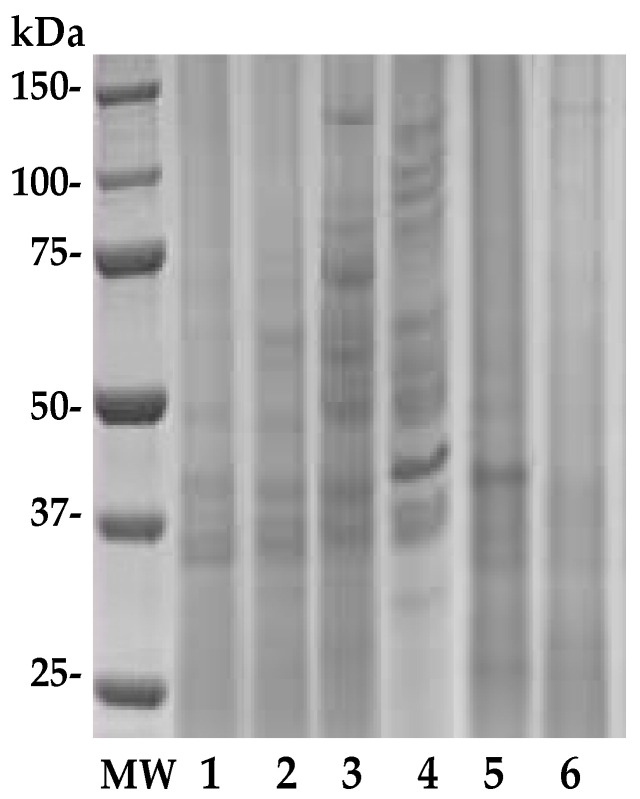
Representative 1-DE gel image. MW, molecular weight marker (All Blue Precision Plus Protein Standard, Bio-Rad) expressed in a kilodalton (kDa). Lane 1, C group-B; lane 2, C group-T; lane 3, CHX group-B; lane 4, CHX group-T; lane 5, CHX+ADS group-B; lane 6, CHX+ADS group-T. Separation was performed on precast gel Bolt 12% Bis-Tris Plus and protein bands were stained with 0.2% silver nitrate.

The proteins isolated from the TSCM samples were first subjected to a 1-DE analysis to evaluate the quality of the protein extracts and the band separation profiles. The obtained results are shown in Figure 1. Protein lanes refer to controls both at basal condition (lane 1) and 4 weeks post-surgery (lane 2), CHX mouthwash therapy in basal situation (lane 3) and 4 weeks after surgery (lane 4) and CHX+ADS before and after 4 weeks of treatment (lane 5 and lane 6, respectively). As evidenced through 1-DE separation and the silver stain method, the 6 groups presented different proteomic profiles. 

The densitometric analysis of the protein bands performed by QuantityOne software, calculated as the sum of the total OD intensity values per lane, is shown in Table 1.

A paired Student’s t-test was used to detect, within the same group, the differences between baseline (B) and post-treatment (T) conditions. In particular, a densitometric analysis of the protein bands was significantly more intense in CHX group-T (mean ± SD; 25,987 ± 3233; *p* < 0.05) compared to CHX group-B (mean ± SD; 13,909 ± 3379) and in C group-T (mean ± SD; 16,250 ± 848, *p* < 0.001) compared to C group-B (mean ± SD; 6400 ± 1221). No significant differences were found between CHX+ADS group-T (mean ± SD; 10,623 ± 3198) and CHX+ADS group-B (mean ± SD; 13,704 ± 4018).

The analysis of variance test (ANOVA) and the Student–Newman–Keuls test (SNK) for multiple comparisons were applied for the data analysis among groups; significant differences in the protein deposition were found when comparing the T-phase between the groups: the CHX group showed the highest deposition (Table 2).

### 3.2. 2-DE Analysis

**Figure 2:**
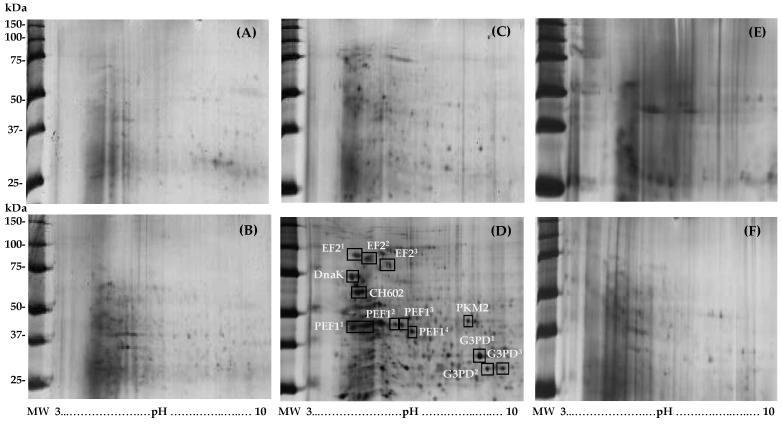
Representative 2-DE protein maps obtained from each pool of TSCM samples. (**A**) C group in the basal condition; (**B**) C group 4 weeks post-surgery; (**C**) CHX group in the basal condition; (**D**) CHX group 4 weeks post-surgery and with mouthwash rinsing; (**E**) CHX+ADS group in the basal condition; (**F**) CHX+ADS 4 weeks post-surgery and with mouthwash treatment. Abbreviated protein names correspond with those reported in Table 3. Bi-dimensional separation was carried out by an IPG strip, pH range 3–10 (first-dimension) and gradient gel 8–16% (second-dimension). The molecular weight marker is expressed in a kilodalton (kDa).

To achieve the detection of a wider proteome coverage, the TSCM samples were further subjected to a 2-DE analysis. In Figure 2 are illustrated the representative 2D protein maps obtained from the TSCM samples of the six groups: (A) C group-B, (B) C group-T, (C) CHX group-B, (D) CHX group-T, (E) CHX+ADS group-B and (F) CHX+ADS group-T. Four weeks after surgery and treatment with CHX mouthwash (panel D) revealed a significant change in the TSCM proteome with respect to the CHX group at the basal state (panel C) in agreement with the results obtained by the 1-DE analysis.

### 3.3. LC-MS/MS Analysis

Differentially expressed protein spots comprised the MW range between 25–150 kDa and were identified in CHX group-T by the LC-ESI-QO-MS/MS system; all the characterized proteins are listed in Table 3.

In the first column, the full name is reported and in column 2 is the abbreviated name of the 13 identified proteins, which correspond with 6 unique proteins: glyceraldehyde-3-phosphate dehydrogenase (G3PD), pyruvate kinase PKM (PKM2), chaperone protein DnaK (DnaK) or heat shock protein 70 (Hsp70), 60 kDa chaperonin 2 (CH602) or heat shock protein 60 (Hsp60), elongation factor 2 (EF2) and putative elongation factor 1-alpha-like 3 (PEF1). For three proteins, G3PD, EF2 and PEF1, different isoforms were detected. The primary accession number, obtained from the NextProt or UniProt databases, are shown in column 3. Moreover, for each identified protein, the experimental MW is indicated along with the highest scores obtained with the MASCOT search engine, the values of significant peptides matching the identified proteins and the significant sequences. Finally, the exponentially modified Protein Abundance Index (emPAI) is reported, which indicates an approximate, label-free, relative quantitation of the proteins in a mixture based on the protein coverage by the peptide matches in a database search result.

## 4. Discussion

The TSCM, collected by curettage, is material that adheres to the middle and incisal thirds of the tooth surface; it should not be given the inappropriate term dental plaque although it is impossible to exclude that part of the TSCM, which may be a structured ecosystem such as dental plaque or calculus. Plaque should not have been present at the collection sites as enrolled patients underwent a professional oral hygiene session prior to surgery and were instructed how to perform proper mechanical plaque control during the 4-week treatment session.

The study performed did not aim to analyze the response of an ecosystem to CHX or CHX+ADS mouthwashes but rather to assess the effects of these on tooth surfaces on which certain molecules may be adsorbed and may undergo a discoloration process.

Protein profile changes in the TSCM induced by three different dental plaque control protocols (tooth brushing combined with placebo rinses or with CHX or CHX+ADS mouthwashes) were analyzed by 1-DE and 2-DE.

By the 1-DE analysis (Figure 1), significant differences in the protein expression, evaluated as an increase in band intensity (OD), were found after treatment when the mechanical control was combined with the placebo (2.5-fold increase) and CHX mouthwash (1.9-fold increase) but not when combined with the CHX+ADS mouthwash. Comparing the three groups after post-surgical treatment, we found statistically significant differences among them. In addition, CHX group-T compared to C group-T and CHX+ADS group-T showed a greater protein deposition; in contrast, CHX+ADS group-T showed less protein deposition (the comparison among groups is shown in Table 2).

The changes revealed in CHX group-T by the 1-DE analysis were confirmed by 2-DE (Figure 2). An LC-MS/MS analysis of the differential spots between CHX group-T and CHX group-B led to the identification of six unique proteins: DnaK, CH602, G3PD, PKM2, EF2 and PEF1 (Table 3). DnaK and CH602 were of a bacterial origin whereas the others were of a human origin; however, it cannot be excluded that they were also of bacterial origin as most bacterial plaque is not represented in the sequence database used for protein identification [31].

DnaK and CH602 belong to the heat shock protein (HSP) or chaperone protein (Cpn) families. Specifically, DnaK is a bacterial chaperone protein belonging to the Hsp70 family (70 kDa heat shock protein), which is highly conserved among all species from bacteria to humans and is involved in many biological and cellular processes under both physiological and stress conditions. The system regulates the heat shock response, the refolding of newly synthesized proteins through binding to nascent polypeptide chains, the assembly and translocation across membranes, the prevention and regulation of the degradation of protein aggregates, the refolding and repair of misfolded proteins and the control of the conformational status of preexisting proteins [32,33]. Bacterial DnaK is a stress-inducible protein that enables cell survival under stress conditions by preventing protein denaturation upon stress [34]. DnaK shares similar functions with CH602 (also called Cpn60 or Hsp60). HSPs are molecular chaperones involved in the folding of emerging proteins and the refolding of denatured ones. Under stressful conditions, these proteins may play a cytoprotective role by intervening in protein repair, the refolding of misfolded peptides and the degradation of irreparable proteins [35].

G3PD and PKM2 are involved in the glycolytic pathway. Bacteria use three main metabolic pathways for the catabolism of glucose including glycolysis. G3PD is a highly conserved enzyme and an essential component of glycolysis, which catalyzes the conversion of glyceraldehyde 3-phosphate into 1,3-bisphosphoglycerate, a reaction in which NAD+ is reduced to NADH. Its role in cellular metabolism and homeostasis is well-defined whereas its function in pathological processes is not well-established. Depending on the cellular context, G3PD may bind several physiologically important proteins and control their activity, thus mediating the cytotoxic or cytoprotective functions [36]. At the same time, G3PD is expressed on the surface of streptococcal cells and is responsible for several interactions such as the binding of *S. pyogenes* to plasmin [37].

PKM2 is an isoform of pyruvate kinase (PK) that catalyzes the transfer of a phosphoryl group from phosphoenolpyruvate to ADP, forming pyruvate and ATP and thus contributing to the control of glycolysis and cellular metabolism [38]. As a glycolytic enzyme, PKM2 predominantly localizes to the cytosol but can translocate to the nucleus to regulate and promote other important non-metabolic functions such as gene transcription, cell cycle progression, cytokinesis and cell proliferation [39].

EF2 has an essential role in the elongation phase of protein synthesis, catalyzing the GTP-dependent ribosomal translocation step during the formation of the acyl bond between the incoming amino acid residue and the peptide chain to form a polypeptide sequence [40]. Under stress conditions or disease, cells replan the protein synthesis to provide an accurate cellular response. Similar to EF2, PEF1 is a GTP-binding protein and an essential component of the translational machinery during the biosynthesis of proteins from mRNA carried out by ribosomes [41].

Changes in the TSCM proteome induced by a mechanical treatment associated with CHX might be a response of the bacterial biofilm to the action of CHX. The up-regulation of glycolytic enzymes, DnaK and CH602, found in CHX group-T could be considered a bacterial response to counteract the denaturing action of CHX. In support of our results and hypothesis, using a 2-DE-MS approach, Steeves et al. reported an increase in the concentration of molecular chaperone proteins (i.e., DnaK) and glycolytic enzymes (such as G3PD) in response to oxidative stress induced in *Fusobacterium nucleatum*, presumably to reduce the amount of reactive oxygen species and their damaging effects [42]. In addition, the increased expression of EF2 and PEF1 may also represent an attempt by the bacterial biofilm to recover CHX-denatured proteins.

Furthermore, the protein modifications detected in CHX group-T could represent a reaction of the bacterial biofilm to the combined activity of the mechanical control and CHX as significant differences were also found in the C group after post-surgical treatment (Table 2). We believe that in the mouth, even in the absence of CHX, Maillard’s reactions produce high molecular weight complexes capable of modifying the surface properties and also increasing the tooth surface roughness, thus facilitating bacterial deposition [43].Unfortunately we did not investigate this aspect by subjecting C group-T to an MS analysis; we are aware that this is a limitation of the study as well as the small sample size and the wide age range. These aspects could be overcome in future research also considering other mouthwashes.

No changes in the TSCM proteome were found in CHX+ADS group-T. The ADS, consisting of ascorbic acid and sodium metabisulphide, interferes with the main processes responsible for dental pigmentation such as protein denaturation (with the formation of metal sulfides) and Maillard’s reaction. It is our opinion, in light of the results obtained, that an ADS could also inhibit the adhesion of CHX to the tooth surface [25], limiting its antiseptic effect: probably, in the presence of an ADS, CHX has less ability to form chemical bonds with the anionic groups (phosphate, sulfate, carboxyl group) present in the bacterial cell wall and this reduces its ability to alter cell permeability. In addition, the inhibition of Maillard’s reactions would limit the tooth surface roughness and this would explain the lower protein deposition in CHX+ADS group-T.

## 5. Conclusions

This is the first study in which the effects on the TSCM proteome after a 4-week use of CHX and CHX+ADS mouthwashes combined with a mechanical control were evaluated. Despite TSCM being a small biological sample with a low protein content, electrophoretic techniques associated with an MS analysis allowed us to reveal significant alterations of its protein profile following the use of CHX. In particular, three major protein systems were found to be altered: (a) glycolytic enzymes, mainly involved in metabolism and cellular homeostasis; (b) HSPs, or molecular chaperones, known as stress proteins, involved in protein maintenance and protective mechanisms (of exclusive bacterial origin); (c) elongation factors, principally involved in protein biosynthesis, the dysregulation of which is associated with disease progression and cellular metabolic changes.

Modifications in the protein profile of the TSCM were not detected in CHX+ADS group-T, presumably due to competition between the ADS and CHX for adhesion to the tooth surface. The ADS also appeared to override the effect of the chemical control on the TSCM proteome. We believe that the obtained results provide a good basis for continuing to investigate the effects of a mechanical control with CHX and CHX+ADS mouthwashes on the TSCM proteome in order to better understand the roles they might play in controlling oral ecosystems.

## Figures and Tables

**Table 1:** Densitometric analysis of the protein bands.

Groups ^(a)^	Total OD Intensity ^(b)^
C group-B	6400 ± 1221
C group-T	16,250 ± 848
CHX group-B	13,909 ± 3379
CHX group-T	25,987 ± 3233
CHX+ADS group-B	13,704 ± 4018
CHX+ADS group-T	10,623 ± 3198

^(a)^ C group-B, control at basal condition; C group-T, control 4 weeks post-surgery; CHX group-B, CHX mouthwash treatment at basal condition; CHX group-T, CHX treatment 4 weeks post-surgery; CHX+ADS group-B, before treatment; CHX+ADS group-T, CHX+ADS mouthwash treatment 4 weeks after surgery. ^(b)^ Sum of the total bands of OD intensity values per lane (expressed as a mean ± SD).

**Table 2:** Densitometric analysis of protein bands: a comparison between the groups at the same stage (basal or post-treatment).

Comparisons Between Groups in Baseline Conditions (B)	Total OD Intensity ^(a)^	*p*-Value
C group-B vs. CHX group-B	6400 ± 1221 vs. 13,909 ± 3379	*p* < 0.05
C group-B vs. CHX+ADS group-B	6400 ± 1221 vs. 13,704 ± 4018	*p* < 0.05
CHX group-B vs. CHX+ADS group-B	13,909 ± 3379 vs. 13,704 ± 4018	*p* ≥ 0.05
**Comparisons Between Groups After Treatment (T)**	**Total OD Intensity ^(a)^**	***p*-Value**
C group-T vs. CHX group-T	16,250 ± 848 vs. 25,987 ± 3233	*p* < 0.05
C group-T vs. CHX+ADS group-T	16,250 ± 848 vs. 10,623 ± 3198	*p* < 0.05
CHX group-T vs. CHX+ADS group-T	25,987 ± 3233 vs. 10,623 ± 3198	*p* < 0.05

**^(a)^** Sum of the total bands of OD intensity values per lane (expressed as a mean ± SD). *p*-values obtained by a Student–Newman–Keuls test (SNK). *p* < 0.05 was considered as statistically significant.

**Table 3:** Protein identification by LC-ESI-QO-MS/MS.

Protein Name	Abbrev. Name ^a^	Acc. Number ^b^	DB	MW ^c^ (kDa)	Score ^d^	Sign. Pept. ^e^	Sign. Seq. ^f^	emPAI ^g^
Glyceraldehyde-3-phosphate dehydrogenase *	G3PD^1^	P04406-1	NextProt	36,201	70	4	2	0.36
Glyceraldehyde-3-phosphate dehydrogenase *	G3PD^2^	P04406-1	NextProt	36,201	60	3	2	0.36
Glyceraldehyde-3-phosphate dehydrogenase *	G3PD^3^	P04406-1	NextProt	36,201	94	3	2	0.23
Pyruvate kinase PKM *	PKM2	P14618-1	NextProt	58,470	44	2	2	0.14
Chaperone protein DnaK ** (or Hsp70)	DnaK	Q5F6W5	SwissProt	68,934	41	6	5	0.31
60 kDa chaperonin 2 ** (or Hsp60)	CH602	Q7NQX1	SwissProt	57,496	61	6	4	0.30
Elongation factor 2 *	EF2^1^	P13639-1	NextProt	96,246	154	6	3	0.13
Elongation factor 2 *	EF2^2^	P13639-1	NextProt	96,246	111	5	5	0.13
Elongation factor 2 *	EF2^3^	P13639-1	NextProt	96,246	62	6	6	0.13
Putative elongation factor 1-alpha-like 3 *	PEF1^1^	Q5VTE0-1	NextProt	50,495	279	8	2	0.25
Putative elongation factor 1-alpha-like 3 *	PEF1^2^	Q5VTE0-1	NextProt	50,495	329	9	2	0.35
Putative elongation factor 1-alpha-like 3 *	PEF1^3^	Q5VTE0-1	NextProt	50,495	280	9	2	0.25
Putative elongation factor 1-alpha-like 3 *	PEF1^4^	Q5VTE0-1	NextProt	50,495	223	8	2	0.25

^a^ Abbreviated protein names. ^b^ Primary accession number from NextProt or SwissProt databases. ^c^ Experimental protein molecular weight. ^d^ The highest scores with the MASCOT search engine. ^e^ Number of significant peptides matching the identified protein. ^f^ Number of significant sequences. ^g^ exponentially modified Protein Abundance Index. Protein of human * or bacterial ** origin.

## Data Availability

All relevant data are within the paper. The datasets used and/or analyzed during the current study are available from the corresponding author on reasonable request.
